# Non‐native pink salmon *Oncorhynchus gorbuscha* carcasses benefit native benthic macroinvertebrates

**DOI:** 10.1111/jfb.70352

**Published:** 2026-02-03

**Authors:** Hui Wei, Gordon H. Copp, Rasmus B. Lauridsen, Tea Bašić, Phil I. Davison, John F. Murphy, James L. Pretty, Michał E. Skóra, Gabriela Zemelka, John Iwan Jones

**Affiliations:** ^1^ School of Biological and Behavioural Sciences, Queen Mary University of London London UK; ^2^ Key Laboratory of Prevention and Control for Aquatic Invasive Alien Species (Ministry of Agriculture and Rural Affairs), Key Laboratory of Alien Species and Ecological Security Pearl River Fisheries Research Institute, Chinese Academy of Fisheries Science Guangzhou China; ^3^ Centre for Environment, Fisheries and Aquaculture Science Lowestoft UK; ^4^ Centre for Ecology, Environment & Sustainability, Bournemouth University Poole UK; ^5^ Environmental & Life Sciences Graduate Program, Trent University Peterborough Ontario Canada; ^6^ Department of Ecology & Vertebrate Zoology Faculty of Biology & Environmental Protection, University of Lodz Łódź Poland; ^7^ Game and Wildlife Conservation Trust, Salmon & Trout Research Centre Dorset UK; ^8^ Krzysztof Skóra Hel Marine Station, Faculty of Oceanography and Geography, University of Gdańsk Hel Poland; ^9^ Present address: Six Rivers Iceland Iceland; ^10^ Present address: Geography, Environment and Planning, School of Health, Medicine and Life Sciences, University of Hertfordshire Hatfield UK

**Keywords:** carcasses, fish invasion, marine‐derived nutrients, North Atlantic region, resource subsidies, stable isotope

## Abstract

The invasion of the North Atlantic by pink salmon *Oncorhynchus gorbuscha* has raised concerns regarding their impact on coastal rivers. Although the influence of marine‐derived nutrients from returning adult *O. gorbuscha* on rivers in their native range has received much attention, the ecological consequences of invasive *O. gorbuscha* for ecosystems outside the native range are largely unknown. To investigate the impact on the density and community structure of benthic macroinvertebrates, *O. gorbuscha* carcasses were added to 12 experimental channels for 60 days at three treatment levels (control, no carcass; low and high, loading rates). Stable isotopes of carbon (δ^13^C) and nitrogen (δ^15^N) were used to determine if nutrients from carcasses were incorporated into native biota. The density of macroinvertebrates increased close to the carcasses in the high‐addition treatment, suggesting aggregation. Furthermore, macroinvertebrates had a higher δ^15^N near to the carcasses in the low‐ and high‐addition treatments after 30 days, indicating uptake from the carcasses. The higher δ^15^N of willow moss *Fontinalis antipyretica* in carcass‐addition treatments indicated that primary producers could also assimilate nutrients from the decomposition of carcasses. Whilst the addition of carcasses resulted in the increased density of small individuals of macroinvertebrates, this did not propagate to changes in community composition in this relatively short experiment. Overall, the results suggest that native biota might benefit from the marine‐derived nutrients transported to streams by invasive *O. gorbuscha*, however, the long‐term effects of such nutrient/energy subsidies on receiving ecosystems require further investigation.

## 1 INTRODUCTION

Invasive fish species frequently alter ecosystem structure and function through adverse interactions with native species, such as predation, competition and habitat displacement (Gallardo et al., 2016). Although invasions by anadromous fishes may result in competition with, or displacement of, native fishes (Hasegawa, 2020), the disturbance of benthic macroinvertebrates due to spawning activity (Moore & Schindler, 2010) and the creation of hypoxia due to mass mortality after spawning (Sergeant et al., 2017), they can import marine‐derived nutrients to upstream sections of river systems thereby facilitating increased productivity of native stream biota (Walsh et al., 2020). Their dual ecological role as both resource subsidies and ecosystem engineers could make the ecological consequence of non‐native anadromous fishes more difficult to predict. For this reason, studies of community‐level impacts could help to elaborate the consequences of the invasion of ecosystems by anadromous fishes.

Pink salmon *Oncorhynchus gorbuscha* (Walbaum 1792) is native to the North Pacific, from the Beaufort Sea to the Sacramento River in North America and from the Laptev Sea (River Lena) in Siberia to the Sea of Japan (River Tumen and Hokkaido) in Asia (Heard, 1991). This species was introduced to northwestern Russia in 1956–1979 (Gordeeva et al., 2015), where hatched fry were released into several coastal rivers around the White and Barents Seas (Mo et al., 2018; Niemelä et al., 2016). These initial introductions were not successful in establishing self‐sustaining populations. However, a subsequent change of stock material (1985–1999) sourced from the more northerly River Ola was successful in establishing *O. gorbuscha* (Niemelä et al., 2016). These established populations are dominated by odd‐year spawners (Gordeeva & Salmenkova, 2011). In recent years, *O. gorbuscha* have been recorded in the coastal rivers of Denmark (including Greenland and the Faroe Islands), Finland, France, Germany, Iceland, Ireland, Norway including Svalbard, Sweden and the UK (ICES, 2024). However, the consequences of the *O. gorbuscha* invasion for the instream community of rivers in the North Atlantic region have received little study (Bonde & Stien, 2024; Dunlop, Eloranta, et al., 2021; Dunlop, Wipfli, et al., 2021).

*Oncorhynchus gorbuscha* is an anadromous species with a typically 2‐year life cycle. The fry emerge from the gravel nest (redds) in spring, migrate to the sea soon thereafter (mass 100–200 mg) and return as mature adults (mass 1–2.5 kg) after c. 12–18 months of feeding in the ocean (Heard, 1991; Lennox et al., 2023). As mass spawning is common and all individuals die after spawning, Pacific salmon carcasses provide important subsidies for freshwater ecosystems in their native range (Walsh et al., 2020). Atlantic salmon *Salmo salar* L. 1758 carcasses also provide important subsidies to the salmonid rivers of the Atlantic coast (Bernthal et al., 2022; McLennan et al., 2019; Williams et al., 2009). However, as *S. salar* do not tend to spawn in such high numbers, and a proportion of individuals do not die after spawning (Fleming, 1998), such nutrient subsidies are far smaller than those provided by Pacific salmonids to the low‐nutrient rivers of their native ranges. Consequently, the carcasses of *O. gorbuscha* bring novel levels of marine‐derived nutrients into rivers in the species' non‐native range, which could be exploited by native biota. In northern Norway, juvenile *S. salar* and brown trout *Salmo trutta* L. 1758 incorporate energy from carcasses and eggs of *O. gorbuscha*, and there is evidence that carcasses can be consumed by terrestrial and semi‐aquatic scavengers (Dunlop et al., 2021, b). Consumption of *O. gorbuscha* eggs by native *S. salar* parr was also noted in the Kola Peninsula, Russia (Rasputina et al., 2016), a phenomenon that is common among Pacific and Atlantic salmonids in their native ranges (e.g. Bailey et al., 2019; Näslund et al., 2015). Nevertheless, in the Pacific northwest, the primary route of entry of matter from salmon carcasses into the food web is through consumption by benthic macroinvertebrates (Kiffney et al., 2018). Knowledge of the impact of *O. gorbuscha* carcasses on benthic food webs remains scarce in their non‐native range.

The rich body of research on the contributions of Pacific salmonid carcasses to benthic food webs and ecosystem productivity in their native range provides a reference base for predicting the consequences of the *O. gorbuscha* invasion of North Atlantic river systems. The abundance and biomass of macroinvertebrates have been found to increase after the experimental addition of salmon carcasses to streams, and macroinvertebrate assemblages differed between streams treated with salmon carcasses and control streams (Kohler & Taki, 2010). Macroinvertebrate assemblages colonizing carcasses were habitat specific, but dominated by Diptera (Rueegg et al., 2014). Whilst such effects could be due to direct consumption of carcasses, the nutrients released from the carcasses can also cause changes. The biomass and nitrogen stable isotope ratios (δ^15^N) of biofilms and periphyton increased in streams with experimental addition of salmon carcasses or carcass analogues (Collins et al., 2016; Richardson et al., 2017). Similarly, the aquatic vascular plants, northern bur‐reed *Sparganium hyperboreum* Laest. ex Beurl. 1852, various‐leaved pondweed *Potamogeton gramineus* L. 1753, and mare's tail *Hippuris vulgaris* L. 1753 were enriched with marine nitrogen in streams naturally supplemented by salmon carcasses (Hicks et al., 2005). Furthermore, marine‐derived nutrients have been incorporated into the terrestrial landscapes surrounding Pacific salmonid rivers resulting in enhanced productivity (Quinn et al., 2018; Rammell et al., 2021). A recent review indicated the relationships between Pacific salmonids and aquatic and terrestrial ecosystem components (density or biomass of biofilm, vegetation, macroinvertebrates, fish and bears) were generally positive or not significant (Walsh et al., 2020). The results from such studies suggest that the consequences of decaying salmon carcasses for biota varied among recipient ecosystems and may depend on the properties of the ecosystem, especially the background nutrient status (Chaloner et al., 2004; Collins et al., 2020; Swain & Reynolds, 2015).

Given the potential impacts of decaying salmon carcasses on benthic stream communities, which are complex and context‐dependent, the aim of the present study was to undertake an initial assessment of the potential effects of decaying carcasses of *O. gorbuscha* on invaded stream food webs. Our specific objectives were to determine if: (1) native macroinvertebrates consume *O. gorbuscha* carcasses, (2) the presence of *O. gorbuscha* carcasses affects the distribution and biomass of native macroinvertebrates, (3) the isotopic composition of native macroinvertebrates changes in a manner consistent with uptake of marine derived nutrients and (4) the elemental composition of native macroinvertebrates changes as a consequence of access to the nutrient‐rich *O. gorbuscha* carcasses. These objectives were addressed in a manipulation experiment, which was conducted in 12 artificial channels fed by and discharging into a nutrient‐rich chalk stream. The experimental channels were divided into controls (no *O. gorbuscha* carcass) and treatments (low and high amounts of *O. gorbuscha* carcasses added). Many rivers around the North Atlantic have been invaded by high numbers of *O. gorbuscha* (Staveley et al., 2025) and the species appears to be establishing in regions far from the source population (Skóra et al., 2023, 2024). Moreover, the River Frome is more productive than the rivers in their native range. The consequences of *O. gorbuscha* carcasses for native biota in invaded nutrient‐rich rivers remain unknown. Thus, this investigation could provide background information on the impact on macroinvertebrates in invaded rivers.

## 2 METHODS

### 2.1 Experimental design

To determine if *O. gorbuscha* carcasses have an impact on benthic food webs, a randomized block experiment was carried out from September to December 2019 where carcasses were added experimentally to open‐air, flow‐through, stream mesocosm channels. Twelve experimental channels (Figure S1) were arranged in four blocks, each comprising three linear‐stream mesocosms (0.33 m in width, 12.40 m in length and 0.30 m in depth), which were positioned ≈ 140° to the in‐flowing water supply, the Mill Stream, a carrier of the nutrient‐rich (nitrate 6.135 mg N l^−1^, total phosphate 69 μg P l^−1^) River Frome, Dorset, UK (WGS84 latitude, longitude: 50.680781, −2.186435). The channel bottoms consisted of 20 cm depth of a mixture of sand, gravel, pebbles and cobbles, which replicated the sediment‐size distribution of local chalk streams (Growns et al., 2017). Natural macroinvertebrate colonization of the channels, by drift from the Mill Stream and by adult oviposition, was complemented by the addition of macroinvertebrates collected from the River Frome by kick sampling using a pond net: the macroinvertebrates were mixed and aliquots added to the head of each channel. The macroinvertebrate communities in the channels are a realistic reflection of those in the River Frome (Ledger et al., 2009). The channels were shaded throughout the colonization period using cloth tarpaulin covers, approx. 60% shading, to ensure that excess diatom growth did not occur.

Frozen carcasses of *O. gorbuscha*, which had been collected from rivers in northeast England in 2017 and verified to be free of pathogens by the Environment Agency, were added to the channels on 18 October 2019. Whilst the density of carcasses may be locally high, particularly if hydrological conditions encourage accumulation, the carcass density treatments were based on loading rates used for other salmon‐related studies in their native range (Bilby et al., 1998; Claeson et al., 2006; Cram et al., 2011; Heintz et al., 2004). Hence, following a randomized block design, three loading rates of carcasses were used, namely control (0 kg m^−2^), low (0.05 kg m^−2^) and high (0.15 kg m^−2^). The carcasses were held in 1 cm aperture mesh bags (0.3 × 0.3 m) and pinned to the bed with steel poles 50 cm from the upstream end of the channel. Empty mesh bags were placed in the control treatment.

The macroinvertebrate community was sampled 0, 30, 45 and 60 days after carcass addition using a Surber sampler (15 × 15 cm, 300 μm mesh net). On each occasion three Surber samples were collected from each channel at different distances from the carcasses: close (within 1 m), moderate (5 m downstream) and far (10 m downstream) from the mesh bags (Figure S1). The samples were immediately frozen (−20°C) for identification later. Macroinvertebrate specimens were identified to the lowest practical taxonomic level (usually species), counted and body parts measured to estimate individual biomass (Woodward et al., 2010). The density, total biomass and mean individual biomass of each taxon were calculated. Macroinvertebrate taxa were classified as their functional group according to Tachet et al. (2002: tab. S1). On sampling occasions 45 and 60 days the mesh bags were carefully lifted, weighed and returned to the head of the channel. As all the soft tissue had gone from the carcasses, with only bones remaining, the experiment was terminated after 60 days.

To determine if *O. gorbuscha* carcasses were incorporated into the benthic food web, macroinvertebrates and benthic resources were collected for stable isotope analysis. Individuals of each macroinvertebrate species from each sample were processed immediately after identification and measuring. The guts were removed by dissection under microscope, and soft tissues of snails and bivalves extracted. Basal resources were also collected from the locations where the macroinvertebrates were sampled. For fine particulate organic matter (FPOM), an area of bed was isolated with a bottomless bucket, the surface layer of the sediment suspended by agitating the water and a sample of the suspension collected in a plastic bottle. Coarse particulate organic matter (CPOM) was collected by hand. Periphyton (algal‐dominated biofilm) was removed from five to six stones with a toothbrush and the suspension retained in plastic bottles. Aquatic plants, principally willow moss *Fontinalis antipyretica* Hedw. 1801, were collected by hand, and vigorously shaken in stream water for 30 s to remove all particulates and attached algae. A sample of the dorsal muscle tissue was collected from each *O. gorbuscha* carcass at the start of the experiment. All samples were frozen at −20°C immediately.

All samples were dried at 60°C for 48 h and individuals ground to powder using a mortar and pestle (for small‐bodied species, two to three individuals were homogenized as one sample). Basal resources were also dried and ground, with the exception of periphyton, which was filtered (Whatman GF/C), dried and sampled with a punch. Material was loaded into individual tin capsules and weighed. Three replicates of each species per location/occasion were combusted and isotope ratios determined using a mass spectrometer system in the continuous‐flow (CF‐IRMS) mode, with a Carlo Erba elemental analyser (CHN 1110) coupled to a Delta Plus mass spectrometer (Thermo Scientific). All values are presented in parts per thousand (‰) using the delta notation (δ), which represents the deviation of stable isotope ratios (^13^C:^12^C and ^15^N:^14^N) from international reference standards: Vienna PeeDee Belemnite limestone for carbon and atmospheric nitrogen for nitrogen. Replicate samples of an internal standard (Casein) yielded results that were precise and accurate, δ^13^C = −26.90 ± 0.09 ‰ (mean ± standard deviation [SD], *N* = 20), δ^15^N = 5.94 ± 0.21 ‰ (mean ± SD, *N* = 20). Lipid normalization was performed for macroinvertebrates using the equation of Post et al. (2007) when C/N ratios were more than 3.5.

### 2.2 Data analysis

To determine the effect of *O. gorbuscha* carcasses on the density and total biomass of macroinvertebrates (i.e. all the macroinvertebrates pooled, hereafter macroinvertebrates), repeated‐measures general linear mixed models were used that included block, carcass additions (Treatment), distance from the carcasses (Distance), time (Occasion) and their interactions. The identity of macroinvertebrate taxa (species) was nested at the lowest level and included as a random factor. To further explore which components of the macroinvertebrate community were affected, the same repeated‐measures general linear mixed model (excluding species) was used to assess influences on the variation in eight macroinvertebrate functional groups and the five most frequent taxa (>200 individuals, i.e. freshwater shrimp *Gammarus pulex* (L. 1758), midges Chironomidae, green drake mayfly *Ephemera danica* Müller 1764, common net spinner *Hydropsyche pellucidula* (Curtis 1834) and water louse *Asellus aquaticus* (L. 1758)). To mitigate the effect of multiple comparisons on type I error, the Holm–Šidàk adjustment was used to establish statistical significance. A further general linear model was used to assess the impact of Block, Distance, Occasion, Treatment and their interactions on the individual mass of macroinvertebrates and of the five most abundant taxa. Replicates of each species were nested within the interaction among Treatment, Distance and Species, and set as a random effect. Differences in individual mass among treatments across time and distance to carcasses were determined by their 95% confidence intervals. The data were checked for normality using the Q‐Q plot method and transformed where necessary. Densities were log_10_(*x* + 1) transformed to meet the assumption of normality. Data analysis was conducted in SAS 8.1.

Partial redundancy analysis was carried out to detect the impact of *O. gorbuscha* carcass addition and sampling occasion on macroinvertebrate community structure, using block as a covariate. Species with low frequencies of occurrence (<3% of samples) were excluded from the analysis. Densities were log_10_(*x* + 1) transformed. As a measure of temporal change, the Euclidean distance between the centroids for the sampling occasions was calculated for control, low and high carcass addition treatments. Data analysis was conducted in Canoco 5.1.

To determine the extent to which *O. gorbuscha* carcasses were incorporated into the benthic food web, and if the elemental composition of native macroinvertebrates changed as a consequence of access to the nutrient‐rich carcasses, the same repeated‐measures general linear mixed model detailed above was used to determine the impact of Block, Treatment, Distance, Occasion and their interactions on the δ^13^C, δ^15^N, %C, %N and C:N of macroinvertebrates and primary producers (*F. antipyretica* and periphyton). δ^15^N was log_10_ transformed to meet the assumption of normality. In each model, where appropriate, the identity of macroinvertebrate species, nested at the lowest level, was included as a random factor. To mitigate the effect of multiple comparisons on type I error, the Holm–Šidàk adjustment was used to establish statistical significance.

Changes in the trophic structure of the macroinvertebrate assemblage following carcass addition were analysed using SIBER in R (Layman et al., 2007). Isotopic niche widths were calculated as standard ellipse area corrected for small sample size for each treatment over the four sampling occasions. To test the hypothesis that the carcasses resulted in a change in the isotopic niche of macroinvertebrates, the extent to which the standard ellipses of the treatments overlapped with that of the control were calculated using the function maxLikOverlap in SIBER.

### 2.3 Ethics Statement

All work was undertaken in compliance with UK animal welfare legislation, the Animals (Scientific Procedures) Act 1986. However, due to the nature of the work, a Home Office licence was not required.

## 3 RESULTS

### 3.1 Density and mass

**FIGURE 1:**
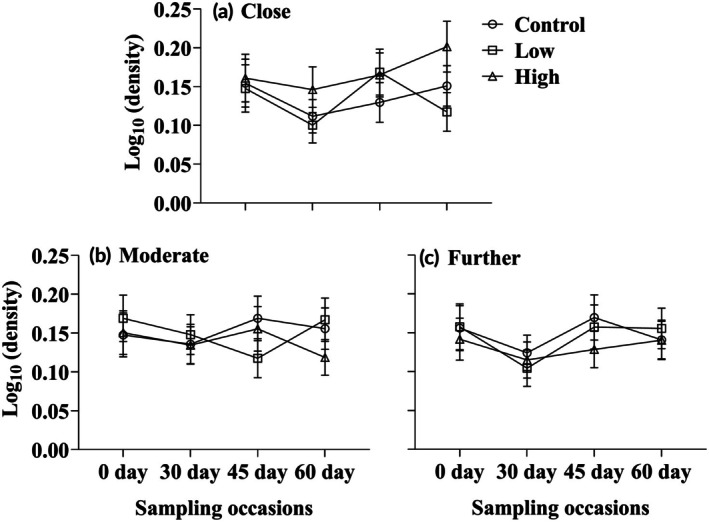
Variation in the density of macroinvertebrates close to (a), moderately far from (b) and far (c) from the pink salmon *Oncorhynchus gorbuscha* carcasses across sampling occasions. Overall sample size *n* = 5184.

The effect of the *O. gorbuscha* carcass additions on the density of macroinvertebrates depended on distance from the carcasses and on sampling occasion (Figure 1). Carcass addition resulted in a higher total density of macroinvertebrates close to the carcasses (Treatment × Distance) and the difference became more pronounced with time (Treatment × Distance × Occasion; Table 1), suggesting aggregation around this novel resource. The density of macroinvertebrates close to the carcasses increased in the high‐addition treatment relative to the control after 30, 45 and 60 days. In contrast, in the low‐addition treatment, the density was increased relative to the control after 45 days, but decreased after 30 and 60 days. No effects of the treatments were seen at moderate and far distances from the carcasses. Whilst visual observation confirmed that many macroinvertebrate species gathered around the carcasses, the effect on the density of individual taxa was only significant for *A. aquaticus* (Table S2). The interactions of Treatment, Distance and Occasion affected the density and total mass of *A. aquaticus*, which were at higher densities and total mass close to the carcass in the high‐addition treatment on day 60 compared with the control.

**TABLE 1:** Results of a repeated‐measures general linear mixed model to assess the influence of block, pink salmon *Oncorhynchus gorbuscha* carcass addition (Treatment), sampling occasion (Occasion), distance from the carcasses (Distance) and macroinvertebrate taxa (Species) on the abundance, total and individual mass of macroinvertebrates in the channels.

Effect	Log density + 1		Log total mass		Individual mass	
	*F* value	*p*	*F* value	*p*	*F* value	*p*
Block	51.93	**<0.0001**	10.98	**<0.0001**	1.32	0.27
Treatment	0.18	0.83	1.07	0.57	4.57	**0.0309**
Distance	0.51	0.60	1.10	0.55	4.33	**0.0391**
Treatment × Distance	3.77	**0.0137**	1.54	0.19	2.60	0.07
Species	266.10	**<0.0001**	66.21	**<0.0001**	47.8	**<0.0001**
Treatment × Species	0.64	0.99	0.91	0.69	2.93	**<0.0001**
Distance × Species	1.94	**<0.0001**	2.05	**<0.0001**	1.55	**0.0046**
Treatment × Distance × Species	0.93	0.91	0.89	0.82	6.61	**<0.0001**
Occasion	6.69	**0.0002**	10.96	**<0.0001**	27.54	**<0.0001**
Treatment × Occasion	0.34	0.92	0.71	0.87	8.46	**<0.0001**
Distance × Occasion	0.73	0.63	1.02	0.65	9.00	**<0.0001**
Treatment × Distance × Occasion	1.98	**0.0447**	1.60	0.09	8.07	**<0.0001**
Species × Occasion	6.15	**<0.0001**	4.48	**<0.0001**	21.34	**<0.0001**
Treatment × Species × Occasion	0.73	0.99	0.60	0.10	10.39	**<0.0001**
Distance × Species × Occasion	0.88	0.88	0.98	0.80	7.49	**<0.0001**
Treatment × Distance × Species × Occasion	0.77	0.99	0.80	0.99	5.33	**<0.0001**

*Note*: Block, treatment, and distance to the carcasses (Distance) were included in the models as fixed effects, and taxon (Species), which was nested at the lowest level, as a random effect. Significant effects are shown in bold.

**FIGURE 2:**
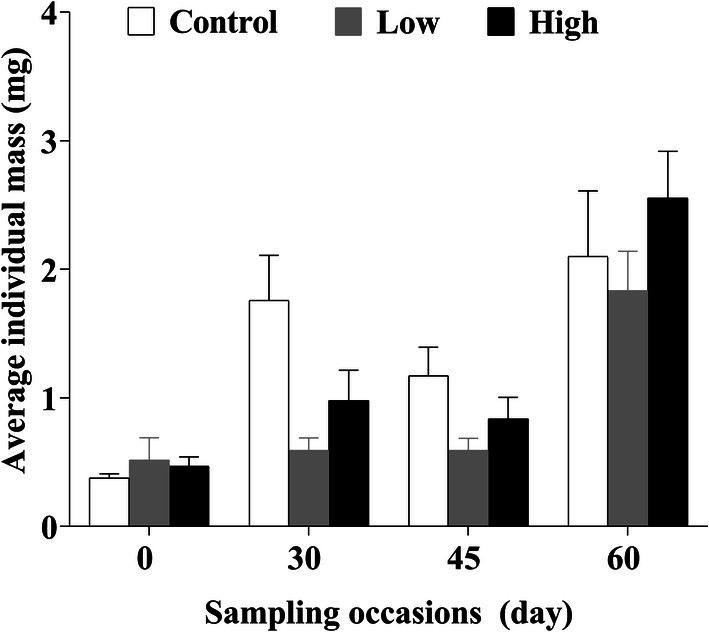
Variation in the average individual mass of macroinvertebrates in response to pink salmon *Oncorhynchus gorbuscha* carcass addition across sampling occasions. Overall sample size *n* = 5184.

**FIGURE 3:**
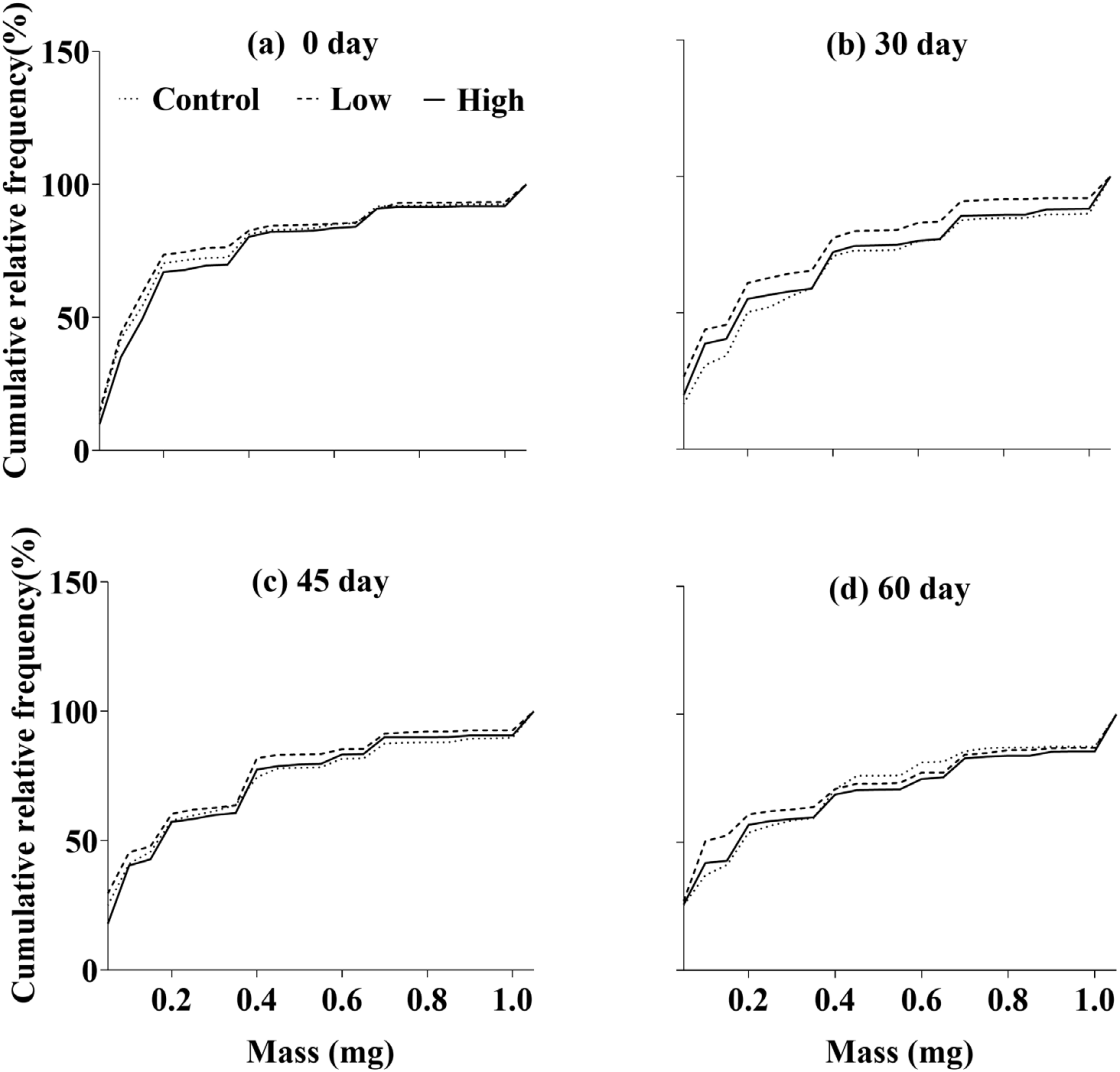
Cumulative relative frequency of individual mass of macroinvertebrates in response to pink salmon *Oncorhynchus gorbuscha* carcass addition on different occasions. Overall sample size *n* = 14,397.

The *O. gorbuscha* carcass additions also appeared to influence the size distribution of macroinvertebrates (Table 1 and Figure 2). The mean individual mass of macroinvertebrates was significantly affected by treatment and the interactions of treatment and occasion, and treatment, distance and occasion. Mean individual mass was higher in the control than in the low‐addition treatment on day 30 (Figure 2). This is consistent with an increased number of small (<0.2 mg), probably young, macroinvertebrates in the carcass‐addition channels (Figure 3b). In contrast, the mean individual mass of macroinvertebrates was highest in the high‐addition treatment on day 60, consistent with an increase in the numbers of large (>1 mg) individuals (Figure 3d). The mean individual mass of *G. pulex* and Chironomidae was significantly affected by the interaction between sampling occasion and carcass addition (Table S3). Mean individual mass of *G. pulex* was higher in the control than in the low carcass‐addition treatment on day 30 (control: mean 0.24 mg, 95% confidence interval 0.21–0.28 mg; low: mean 0.19 mg, 95% confidence interval 0.17–0.21 mg). Mean individual mass of Chironomidae was higher in the control than in the low carcass‐addition treatment on day 45 (control: mean 0.36 mg, 95% confidence interval 0.32–0.41 mg; low: mean 0.25 mg, 95% confidence interval 0.20–0.31 mg).

**FIGURE 4:**
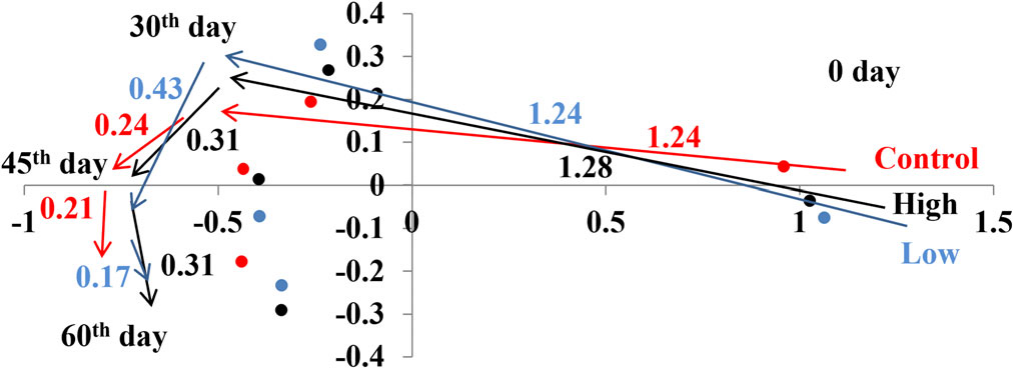
Partial redundancy analysis of the impact of pink salmon *Oncorhynchus gorbuscha* carcass additions on the macroinvertebrate community in experimental channels 0, 30, 45 and 60 days after carcass loading. The Euclidean distance corresponding to change in the centroids of control, low and high carcass addition treatments across the four sampling occasions is presented beside the arrows. Red symbols, control; blue symbols, low carcass addition; black symbols, high carcass addition. Overall sample size *n* = 7344.

Partial redundancy analysis indicated that the macroinvertebrate community varied with sampling occasion (Figure 4 and Table S4). Although treatment had little influence on macroinvertebrate community composition, the community in the control moved a shorter Euclidean distance (1.69) than those in the low (1.84) and high (1.90) carcass addition treatments, and the high‐addition treatment had the longest change distance between each sampling occasion.

### 3.2 Isotopic and elemental composition

**FIGURE 5:**
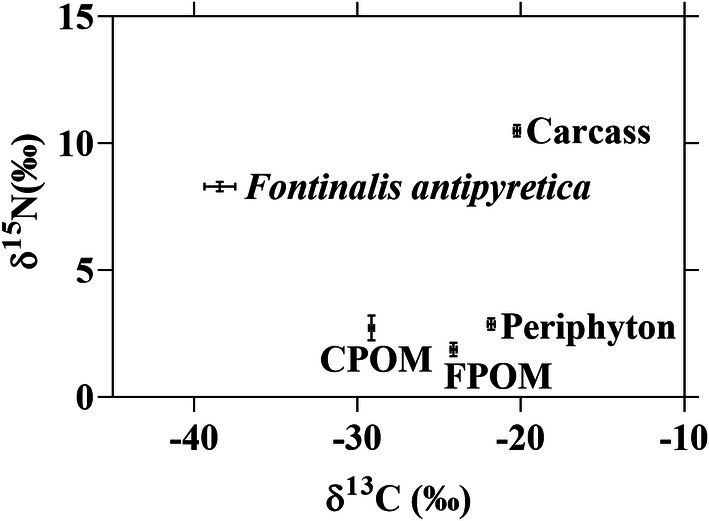
Isotopic composition of basal resources in the control treatment and pink salmon *Oncorhynchus gorbuscha* carcasses. Symbols indicate the mean and standard deviation of δ^13^C and δ^15^N. Overall sample size = 419. CPOM, coarse particulate organic matter; FPOM, fine particulate organic matter.

The isotopic composition of *O. gorbuscha* carcasses differed substantially from the other basal resources in the channels (Figure 5). *Oncorhynchus gorbuscha* carcasses had a substantially higher δ^15^N than all other basal resources, and a less negative δ^13^C than *F. antipyretica*, although δ^13^C was less different from CPOM, FPOM and periphyton (Figure 5). Hence, uptake of material from the carcasses would be expected to have a larger influence on δ^15^N than on δ^13^C.

After 45 days, 92.1% of the soft tissue of the carcasses had been lost in the low‐addition treatment and 87.0% in the high‐addition treatment. Effects of the carcass addition treatments were seen in the isotopic ratios of macroinvertebrates. Whilst Treatment had a significant overall effect on δ^15^N (Table 2), there were spatial (Block, Distance) and temporal (Occasion) differences in isotopic ratios (Table S5). The carcass‐addition treatments had effects on the isotopic ratios of both elements, which took time to develop (Treatment interactions with Occasion). This was expected as the macroinvertebrates would take time to assimilate the carcass tissues (Table 2). In this respect, the δ^15^N of macroinvertebrates was significantly higher after 30 days in the low and high‐addition treatments (control: mean ± se δ^15^N = 8.87 ± 0.11; low: δ^15^N = 9.05 ± 0.11; high: δ^15^N = 9.09 ± 0.10) and δ^13^C was lower after 30 days in the high‐addition treatment than in the control (control: mean ± se δ^13^C = −30.68 ± 0.24’ high: δ^13^C = −31.27 ± 0.24).

**TABLE 2:** Results of repeated‐measures general linear mixed models assessing the impacts of pink salmon *Oncorhynchus gorbuscha* carcass‐addition (Treatment) on the stable isotope ratios of carbon (δ^13^C) and nitrogen (δ^15^N) of all macroinvertebrates collectively, macroinvertebrate functional groups (absorber, deposit feeder, filter feeder, piercer, predator, scraper and shredder) and primary producers (*Fontinalis antipyretica* and periphyton).

	Isotope	Treatment	Treatment*distance	Treatment*occasion	Treatment*distance*occasion
Macroinvertebrates					
Macroinvertebrates	δ^13^C	0.48	2.06†	4.58***	3.28***
	δ^15^N	13.07***	2.58*	2.96**	3.43***
Absorber	δ^13^C	1	0.15	2.28	/
	δ^15^N	0.73	1.34	1.59	/
Deposit feeder	δ^13^C	1.78	0.73	1	1.07
	δ^15^N	16.81***	4.1*	21.85***	15.44***
Filter feeder	δ^13^C	9.91***	1.47	0.77	5.48***
	δ^15^N	1.26	1.83	1.8	2.05
Piercer	δ^13^C	1.4	0.8	0.46	3.98*
	δ^15^N	2.99	0.67	0.78	1.7
Predator	δ^13^C	5.90*	2.07	1.62	0.49
	δ^15^N	0.38	0.15	0.85	2.87
Scraper	δ^13^C	2.21	0.83	1.01	0.71
	δ^15^N	5.08*	0.32	2.92†	1.69
Shredder	δ^13^C	0.5	1.35	1.74	1.67
	δ^15^N	1.46	2.11	0.79	1.81
Primary producers					
*Fontinalis antipyretica*	δ^13^C	3.12†	0.13	3.03*	0.11
	δ^15^N	4.72*	0.11	1.92	0.34
Periphyton	δ^13^C	7.98**	0.16	4.25**	0.17
	δ^15^N	0.55	1.42	0.71	0.7

*Note*: Block, Treatment, distance from the carcasses (Distance) and sampling occasion (Occasion) were included in the models as fixed effects, and taxon, which was nested at the lowest level, as a random effect. *F* values are presented with significant results indicated as ***p ≤ 0.001, **p ≤ 0.01 and *p ≤ 0.05. Marginal results (*p* ≤ 0.1) are indicated as †. Missing values are due to unbalanced sample sizes. Full details of all main effects and interactions are in Table S3.

The effect of the carcass‐addition treatments on the isotopic ratios differed among macroinvertebrate functional groups, with significant effects seen in deposit feeders, filter feeders, piercers and predators that tended to be localized and take time to develop (Treatment interactions with Occasion and Distance; Table 2). The δ^15^N of deposit feeders was significantly affected by Treatment and its interactions with Occasion and Distance (Table S5). The effect of the carcass addition treatments also differed among taxa, influencing the δ^13^C of *A. aquaticus*, close to the carcasses (Treatment × Distance; Table S6). However, it should be noted that the reduced number of individuals per taxon available per sample at this higher resolution had an influence on the power to detect differences.

The treatments also affected the primary producers, an effect that, again, took time to develop, consistent with the release of nutrients and particulate material from the carcasses (Table S5). The δ^13^C of periphyton was marginally lower in the high‐addition treatment (control: mean ± se δ^13^C = −21.81 ± 0.21; high: δ^13^C = −22.30 ± 0.27; *t* = 2.23, corrected *p* = 0.06). Periphyton %C was higher in the high‐addition treatment than in the control (control: mean ± se %C = 19.28 ± 0.47; high: %C = 21.35 ± 0.63; *t* = −3.18, corrected *p* = 0.006; Table S7). This result was consistent with a negative correlation between δ^13^C and %C of periphyton (Spearman correlation coefficient = −0.321, *p* = 0.01). The δ^15^N of *F. antipyretica* was significantly higher in both the carcass addition treatments (control: mean ± se δ^15^N = 8.30 ± 0.21; low: δ^15^N = 8.98 ± 0.13, *t* = −2.69, corrected *p* = 0.03; high: δ^15^N = 8.91 ± 0.20, *t* = −2.53, corrected *p* = 0.04; Table S5), whereas the δ^13^C of *F. antipyretica* was lower in the low and high addition treatments after 45 days than in the control (control δ^13^C = −36.82 ± 1.72; low: δ^13^C = −42.10 ± 1.08, *t* = 3.63, corrected *p* = 0.02; high: δ^13^C = −42.96 ± 0.42, *t* = 3.79, corrected *p* = 0.01; Table S5).

The carcass‐addition treatments also affected the elemental composition of the macroinvertebrates when considered all together or as separate functional groups, and again the effect took time to develop (Treatment interactions with Occasion; Table S7). The interaction of Treatment and Occasion had a significant effect on %C of shredders. Finally, the interaction of Treatment, Distance and Occasion had a significant effect on the %N of filter feeders, the %C and %N of shredders, and a close to significant effect on %C filter feeders and %N of scrapers (Table S7).

**FIGURE 6:**
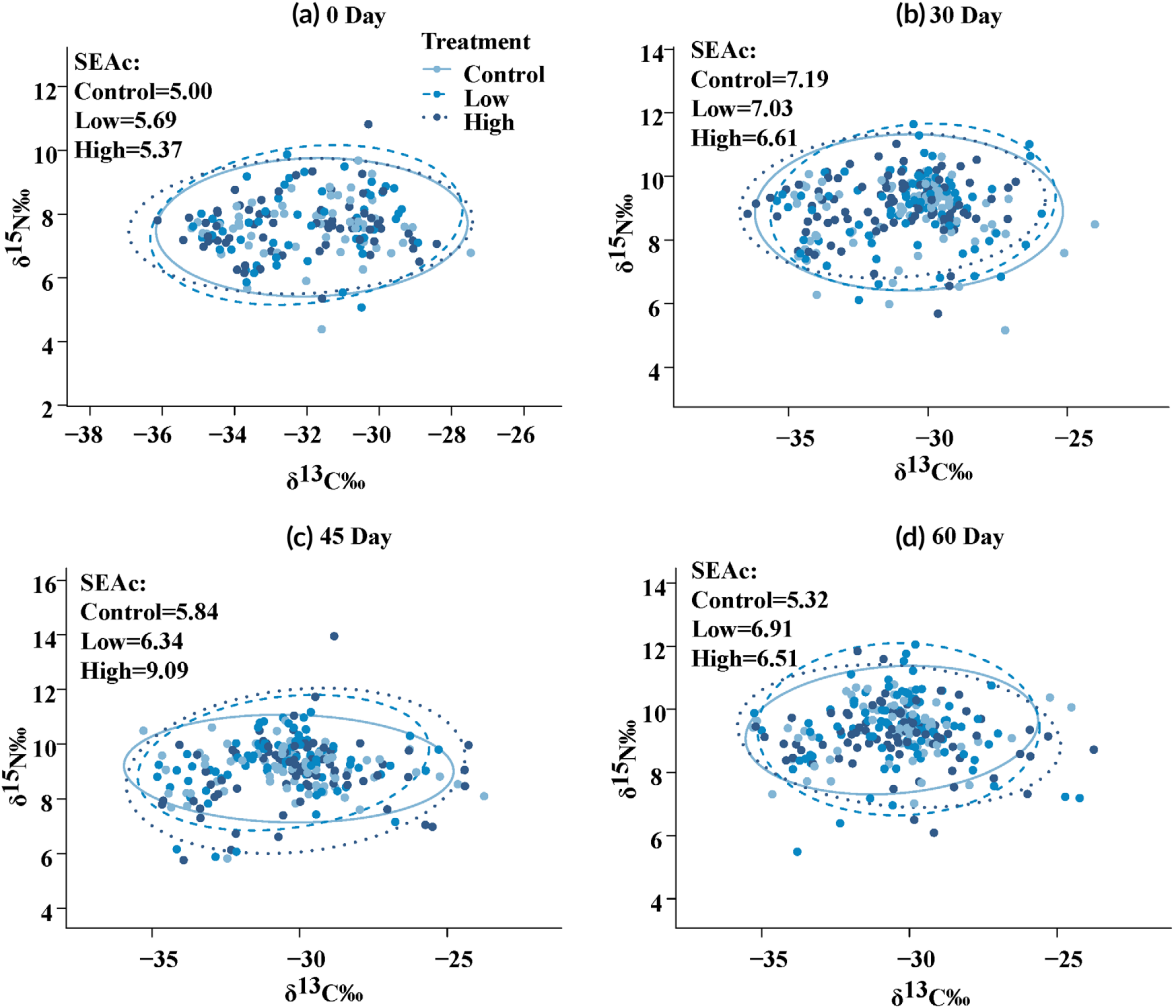
Isotopic niches of macroinvertebrates from control, low and high carcass addition treatments on different sampling occasions, area determined as standard ellipses corrected for small sample size (SEAc). For details of extent of overlap between treatments and control see Table 3. Overall sample size = 919.

The isotopic niche of macroinvertebrates varied across sampling occasions. After 45 and 60 days macroinvertebrates had a wider isotopic niche in the low‐ and high‐addition treatments than in the control (Figure 6). The extent to which the isotopic niche of the macroinvertebrates in the carcass addition treatments overlapped with that in the control changed over time, with a reduced overlap of treatment on control but a high overlap of control on treatment after 45 and 60 days (Table 3). This change in overlap is indicative of an expansion of the isotopic niche and was most pronounced in the high treatment on day 45.

**TABLE 3:** Summary of the percentage of overlap in isotopic niches of macroinvertebrates in the carcass addition treatments and the control.

	Overlap of low on control	Overlap of control on low
Day 0	85.90%	97.81%
Day 30	94.08%	91.96%
Day 45	81.52%	88.60%
Day 60	75.22%	97.74%

	Overlap of high on control	Overlap of control on high
Day 0	91.19%	97.92%
Day 30	91.09%	85.68%
Day 45	63.54%	99.00%
Day 60	79.96%	97.94%

*Note*: Percentages of overlap were calculated in a pairwise fashion using the standard ellipse area. Extensive (close to 100%) or minimal (close to 0%) overlap was considered indicative of a substantial similarity or difference, respectively. A lower overlap of treatment on control coupled with a high overlap of control on treatment is indicative of an expansion of the isotopic niche.

## 4 DISCUSSION

In their native range, nutrients from Pacific salmon carcasses are incorporated into food webs via various pathways (Kiffney et al., 2018), with contributions to macroinvertebrates being an important pathway (Hicks et al., 2005). Our experiment using the carcasses of invasive, non‐native *O. gorbuscha* indicates that native European macroinvertebrates similarly exploit this novel resource. Macroinvertebrates exposed to carcasses had a higher δ^15^N, indicating that matter from *O. gorbuscha* carcasses was incorporated into their tissues, with the effect developing over time and close to the carcasses. This is consistent with the increase in the density of macroinvertebrates close to the carcasses, suggesting that the macroinvertebrates aggregated around this novel resource and exploited it. Yet, the effect of the carcass addition was not restricted to higher trophic levels. The higher δ^15^N of *F. antipyretica* in the carcass addition treatments indicated that primary producers could assimilate the soluble nutrients liberated from the decomposition of carcasses. These results suggest native biota might benefit from the marine‐derived nutrients transported to rivers by invasive *O. gorbuscha*. Whilst novel, these findings are not surprising: the marine nutrients transported by *O. gorbuscha* are known to be of critical importance for nutrient‐poor rivers in their native range (Kohler & Taki, 2010; Walsh et al., 2020). Here we have demonstrated experimentally, and under relatively nutrient‐rich conditions, that the marine resources delivered by non‐native *O. gorbuscha* can impact benthic communities in their invaded range.

In nature, the flesh, skin, eggs, bones and biofilm growing on salmon carcasses can be directly consumed by consumers, depending on their feeding mode and mouth size (Cummins & Klug, 1979; Gende et al., 2002). The dissolved nutrients released through the decomposition of carcasses and excreta of consumers may also be utilized by primary producers, i.e. periphyton, aquatic plants and riparian vegetation (Hicks et al., 2005). Detrital resources may also change through the release of particulate material either directly from the carcasses or as faeces from consumers, which in turn will affect the isotopic composition and density of detritivores, as seen in our findings. These subsidies, derived from various pathways, can affect trophic interactions within the food webs of receiving systems (Collins et al., 2020).

The isotopic niche of macroinvertebrates increased in the presence of carcasses, which indicates an increase in trophic diversity as macroinvertebrates incorporated carcasses into their tissues. In the native range of *O. gorbuscha*, the dominant taxa that feed on carcasses are Baetidae, Chironomidae and Heptageniidae, which mainly consume organic particles and detritus (Chaloner et al., 2002; Morley et al., 2016). In the present study, of all the functional feeding groups, deposit feeders appeared to benefit most clearly from the presence of the carcasses, with a change in δ^15^N in carcass‐addition treatments and especially close to carcasses. Nevertheless, the dominant species in the experimental channels was the shredder *G. pulex*, a detritivore that usually feeds on leaf litter (Consolandi et al., 2021). Yet, carcass addition had no apparent effect on *G. pulex* densities, even close to the carcasses. Although the diet preference of the other abundant shredder in the channels, *A. aquaticus*, is similar to *G. pulex* (i.e. detritus, leaf litter; Tachet et al., 2002), *A. aquaticus* were affected by the addition of carcasses, both isotopically (δ^13^C) and being more abundant in the high‐addition channels, particularly near the carcasses. It is possible that *A. aquaticus* benefitted more from the carcasses, as extra food sources, due to competition from the more abundant *G. pulex* (i.e. Graça et al., 1994). These results suggest that carcasses could be opportunistically consumed by shredders, especially in highly competitive conditions (Corrigan et al., 2011). Although effects on δ^15^N and δ^13^C were observed in some functional groups and species, the changes in density of macroinvertebrates at the species level was not consistent with their patterns of enrichment, which suggests either that we lacked statistical power at the species level or the stable isotope signal of *O. gorbuscha* carcass might not emerge in all species of macroinvertebrates. Macroinvertebrates may benefit from other biologically important minerals and lipids released by the carcasses (Gende et al., 2002). This hypothesis could be supported by the increase in the variety of basal resources (i.e. δ^13^C range) following carcass addition. Alternatively, it may take time for species that do not usually feed on dead animal tissues to become familiar with the novel food (Alexander et al., 2022; Skein et al., 2018).

Besides impacts on macroinvertebrates, the addition of *O. gorbuscha* carcasses impacted basal resources. The higher δ^15^N of *F. antipyretica* in the carcass‐addition treatments indicated that the dissolved nutrients from carcass decomposition could be assimilated by the primary producers (Hicks et al., 2005). The decrease in δ^13^C of periphyton in carcass‐addition treatments could be explained by increased productivity in nutrient‐rich conditions, which would result in less discrimination against ^13^C (Camilleri & Ozersky, 2019). This fact was partly supported by an increase in %C of periphyton in the carcass‐addition treatments, suggesting an increase in the products of photosynthesis. This finding is consistent with Collins et al. (2016), who demonstrated that the standing crop of periphyton increased in streams to which salmon carcasses had been added. Collectively, the results of the present experiment suggest that nutrients from *O. gorbuscha* carcasses were used by native biota through both consumption and mineralisation.

However, due to the short duration of our experiment, the longer‐term implications of repeated inputs of marine‐derived nutrients from invasive *O. gorbuscha* carcasses for native benthos are not as clear. Under our experimental conditions, the effect on community composition was limited and the isotopic effects appeared to be short lived due to the quick isotopic turnover of the macroinvertebrates (Thomas & Crowther, 2015). The effect on the δ^15^N of deposit feeders appeared to become diluted over time, possibly through feeding on ^15^N‐depleted food resources (i.e. particulate organic matter and sediment organic matter) after the carcasses were exhausted. Animals may exhibit compensatory feeding behaviour, increasing the intake of low‐quality diets to compensate for the nutritional deficiency after switching from high‐nutrient food (Slansky & Wheeler, 1992). Shredders were enriched in terms of %C close to the carcasses after 60 days, suggesting an increase in lipid content following consumption of pink salmon tissues, which might indicate allocation of energy to storage to support reproduction in the adult stage. This is consistent with the increase in small individuals of the more abundant taxa found in the experiment, *G. pulex* and Chironomidae. A reproductive response of macroinvertebrates to increased food availability is not surprising: nutrient addition experiments with aquatic gastropods resulted in shorter generation times and increased reproduction in response to increased availability of food (Jones et al., 1999). Furthermore, both *G. pulex* and Chironomidae show flexibility in the number of generations per year depending on temperature and nutrient availability (Graça et al., 1994; Prat & Rieradevall, 1995).

Nutrient subsidies from salmon carcasses can facilitate the growth and recruitment of consumers (Keeley et al., 2016; Wipfli et al., 2003). In the present study, an increase in macroinvertebrate density close to the carcasses and the increased abundance of smaller (younger) size classes of macroinvertebrates suggests both aggregation and increased production. It is likely that the growth and fecundity of macroinvertebrates increased where they had access to the high‐quality food source provided by the carcasses (Gende et al., 2002). Although only minor changes in community composition were seen here, this could simply be a consequence of the relatively short duration of the experiment, as there would be a time lag for any changes in the fecundity of benthic macroinvertebrates to manifest (i.e. Collins et al., 2016). Community composition in the carcass addition treatments changed more than the controls across time, suggesting that the communities could be on different trajectories where the novel nutrient pulse provided by *O. gorbuscha* carcasses could have knock‐on effects in terms of macroinvertebrate fecundity (Robertson & Hutto, 2006). Whilst this novel, non‐native subsidy appeared to benefit the macroinvertebrates, it may take repeated inputs of marine‐derived nutrients from invasive *O. gorbuscha* spawning events before pronounced changes are seen in the macroinvertebrate communities of invaded rivers.

Despite being of relatively short duration and at high background nutrient concentrations, our experimental findings suggest that *O. gorbuscha* carcasses can exert significant effects on the density and assemblage of macroinvertebrates in invaded streams. The response intensity in natural streams is likely to be affected by the density and proximity of carcasses (Claeson et al., 2006; Cram et al., 2011) and background nutrient concentrations. We expect stronger responses of macroinvertebrates in low‐nutrient rivers and especially where the population of invasive *O. gorbuscha* is high.

Although the macroinvertebrate community did not change substantially in the short term of the experiment, growth and fecundity of native macroinvertebrates appears to be affected by consuming carcasses. Over the longer term the effects of *O. gorbuscha* carcasses would be expected to propagate through the food web: increases in the productivity of primary producers and density of invertebrates have been documented following carcass additions in their native range (e.g. Joseph et al., 2020; Kohler et al., 2012). Furthermore, native fish species have been documented as feeding on invasive *O. gorbuscha* eggs (Dunlop, Eloranta, et al., 2021), increasing the benefit at the top of the food web. These findings suggest that native aquatic animals can benefit from the nutrient subsidies of the non‐native *O. gorbuscha* at a local scale, and that such effects might propagate to a larger spatial scale (i.e. river). Nevertheless, native anadromous species (e.g. sea lamprey *Petromyzon marinus* L. 1758, river lamprey *Lampetra fluviatilis* (L. 1758), *S. salar*, *S. trutta* and Arctic charr *Salvelinus alpinus* (L. 1758)), might contribute marine‐derived nutrients to systems, making detection of the effects of *O. gorbuscha* more challenging. Further, preferably long‐term, studies are needed from sites with definitive observations of *O. gorbuscha* spawning to distinguish the effects of their carcasses on native freshwater biota from other effects.

In conclusion, the present study provides new evidence that the invasion of *O. gorbuscha* could be introducing a new source of nutrients into the invaded ecosystems, and that native macroinvertebrates and aquatic plants might benefit from these marine‐derived nutrients. Indeed, even in the nutrient‐rich River Frome, macroinvertebrates appeared to exploit the added *O. gorbuscha* carcasses and benefit from this novel resource. The effect of this novel nutrient source may be short term, such as aggregation around carcasses, but there appears to be a potential lasting effect through increased reproduction of macroinvertebrates. Whilst *O. gorbuscha* may be detrimental for native fish species (e.g. through direct aggression or competition for space), it is possible that there may be some benefits, with *O. gorbuscha* providing marine subsidies that have been lost through declines in native migratory species. The overall consequences of *O. gorbuscha* invasions, both at local and ecosystem levels, urgently requires further study, particularly as many salmonid rivers in the invaded region are naturally nutrient poor. Moreover, the impact of carcass additions on the macroinvertebrate community was not ubiquitous, suggesting that colonization of carcasses might be affected by interspecific competition within the macroinvertebrate assemblage as well as carcass persistence in the stream (Walsh et al., 2020).

## AUTHOR CONTRIBUTIONS

Conceptualisation: H.W., J.I.J., R.B.L. and G.H.C. Developing methods: H.W., J.I.J., J.F.M., R.B.L. and G.H.C. Preparation of figures and tables: H.W. and J.I.J. Conducting the research, data interpretation, and writing: H.W., G.H.C., R.B.L., T.B., P.I.D., J.F.M., J.L.P., M.E.S., G.Z. and J.I.J.

## FUNDING INFORMATION

This study was supported financially by the China Scholarship Council (Grant No. 201903260010), the Central Public‐Interest Scientific Institution Basal Research Fund, CAFS (Grant No. 2020GH04) and the Game & Wildlife Conservation Trust. The participation of G.H.C., P.I.D. and T.B. was supported by the Cefas' Science Excellence fund and the EU INTERREG Atlantic Area funded project “DiadES”. M.E.S. received support from the National Science Centre of Poland (Research activity no. 2018/02/X/NZ8/02252). The study also received support from Queen Mary University of London.

## CONFLICT OF INTEREST STATEMENT

We declare there is no conflict of interest.

## Supporting information


**FIGURE S1.** Schematic diagram of the experimental channels showing one block of three channels. Three random surber samples were collected from each on each occasion, either close (1 m), moderate (5 m) or far (10 m) from the mesh bag containing either control (0 kg m^−2^), low (0.05 kg m^−2^) or high (0.15 kg m^−2^) loading of carcasses. Four blocks were used, comprising 12 channels in total.
**TABLE S1.** Macroinvertebrate taxa collected in the channel experiment. Specimens were identified to the lowest practical taxonomic level (usually species).
**TABLE S2.** Results of repeated measures general linear mixed model for the impacts of carcasses addition on density and total mass of different functional group and dominant species (*n* > 200). ***p ≤ 0.001, **p ≤ 0.01, *p ≤ 0.05 and †*p* ≤ 0.1. DIS, Distance; OCA, Occasion; TRT, Treatment.
**TABLE S3.** Results of the repeated measures general linear mixed model for the impact of Block, Distance, Occasion and Treatment, and their interactions on individual mass of macroinvertebrates and the five most abundant species. ***p ≤ 0.001, **p ≤ 0.01, *p ≤ 0.05 and †*p* ≤ 0.1. DIS, Distance; OCA, Occasion; TRT, Treatment.
**TABLE S4.** Result of partial redundancy analysis of the impact of pink salmon carcass addition and sampling occasion on the community structure of macroinvertebrates.
**Table S5.** Results of repeated‐measures general linear mixed models assessing the impacts of pink salmon carcass addition (Treatment) on the stable isotope ratios of carbon (δ^13^C) and nitrogen (δ^15^N) of all macroinvertebrates collectively, macroinvertebrate functional groups (absorber, deposit feeder, filter feeder, piercer, predator, scraper and shredder) and primary producer (*Fontinalis antipyretica* and periphyton). Block, treatment, sampling occasion (Occasion) and distance from the carcasses (Distance) were included in the models as fixed effects and taxon, which was nested at the lowest level, as a random effect. *F* values are presented with significant results indicated as *** *p* ≤ 0.001, ** *p* ≤ 0.01, * *p* ≤ 0.05 and † *p* ≤ 0.1. Missing values are due to unbalanced sample sizes. DIS, Distance; OCA, Occasion; TRT, Treatment.
**TABLE S6.** General linear model results for the impacts of pink salmon carcass addition (Treatment) on the stable isotope values of carbon (δ^13^C) and nitrogen (δ^15^N) of the most abundant five species. Block, sampling occasion (Occasion) and distance to the carcasses (Distance) were included in the models. Significant effects in bold. *** *p* ≤ 0.001, ** *p* ≤ 0.01 and * *p* ≤ 0.05. Missing values are due to unbalanced sample sizes. DIS, Distance; OCA, Occasion; TRT, Treatment.
**TABLE S7.** Repeated‐measures general linear mixed model results for assessing the impacts of pink salmon carcass addition (Treatment) on elemental content (%C and %N) and ratio (C:N) of macroinvertebrates, functional groups and basal resources in the experimental channels. Block, treatment and distance from the carcasses (Distance) were included in the models as fixed effect and taxon, which was nested at the lowest level, as a random effect. *F* values are presented with significant results indicated as *** *p* ≤ 0.001, ** *p* ≤ 0.01, * *p* ≤ 0.05 and † *p* ≤ 0.1. Missing values for the interaction term were indicated the values were not calculated due to unbalanced sample sizes. DIS, Distance; OCA, Occasion; TRT, Treatment.

## Data Availability

The data are available from the corresponding author upon request.
