# Supplementary material for: Non‐native pink salmon Oncorhynchus gorbuscha carcasses benefit native benthic macroinvertebrates
Source: J Fish Biol. 2026 Feb 3;108(6):1825–37. doi: 10.1111/jfb.70352 (PMC13357299; doi:10.1111/jfb.70352)
Supplement: Supplementary file 1 — FIGURE S1. Schematic diagram of the experimental channels showing one block of three channels. Three random surber samples were collected from each on each occasion, either close (1 m), moderate (5 m) or far (10 m) from the mesh bag containing either control (0 kg m−2), low (0.05 kg m−2) or high (0.15 kg m−2) loading of carcasses. Four blocks were used, comprising 12 channels in total. TABLE S1. Macroinvertebrate taxa collected in the channel experiment. Specimens were identified to the lowest practical taxonomic level (usually species). TABLE S2. Results of repeated measures general linear mixed model for the impacts of carcasses addition on density and total mass of different functional group and dominant species (n > 200). ***p ≤ 0.001, **p ≤ 0.01, *p ≤ 0.05 and †p ≤ 0.1. DIS, Distance; OCA, Occasion; TRT, Treatment. TABLE S3. Results of the repeated measures general linear mixed model for the impact of Block, Distance, Occasion and Treatment, and their interactions on individual mass of macroinvertebrates and the five most abundant species. ***p ≤ 0.001, **p ≤ 0.01, *p ≤ 0.05 and †p ≤ 0.1. DIS, Distance; OCA, Occasion; TRT, Treatment. TABLE S4. Result of partial redundancy analysis of the impact of pink salmon carcass addition and sampling occasion on the community structure of macroinvertebrates. Table S5. Results of repeated‐measures general linear mixed models assessing the impacts of pink salmon carcass addition (Treatment) on the stable isotope ratios of carbon (δ13C) and nitrogen (δ15N) of all macroinvertebrates collectively, macroinvertebrate functional groups (absorber, deposit feeder, filter feeder, piercer, predator, scraper and shredder) and primary producer (Fontinalis antipyretica and periphyton). Block, treatment, sampling occasion (Occasion) and distance from the carcasses (Distance) were included in the models as fixed effects and taxon, which was nested at the lowest level, as a random effect. F values are presented with significant results indi [file JFB-108-1825-s001.docx]

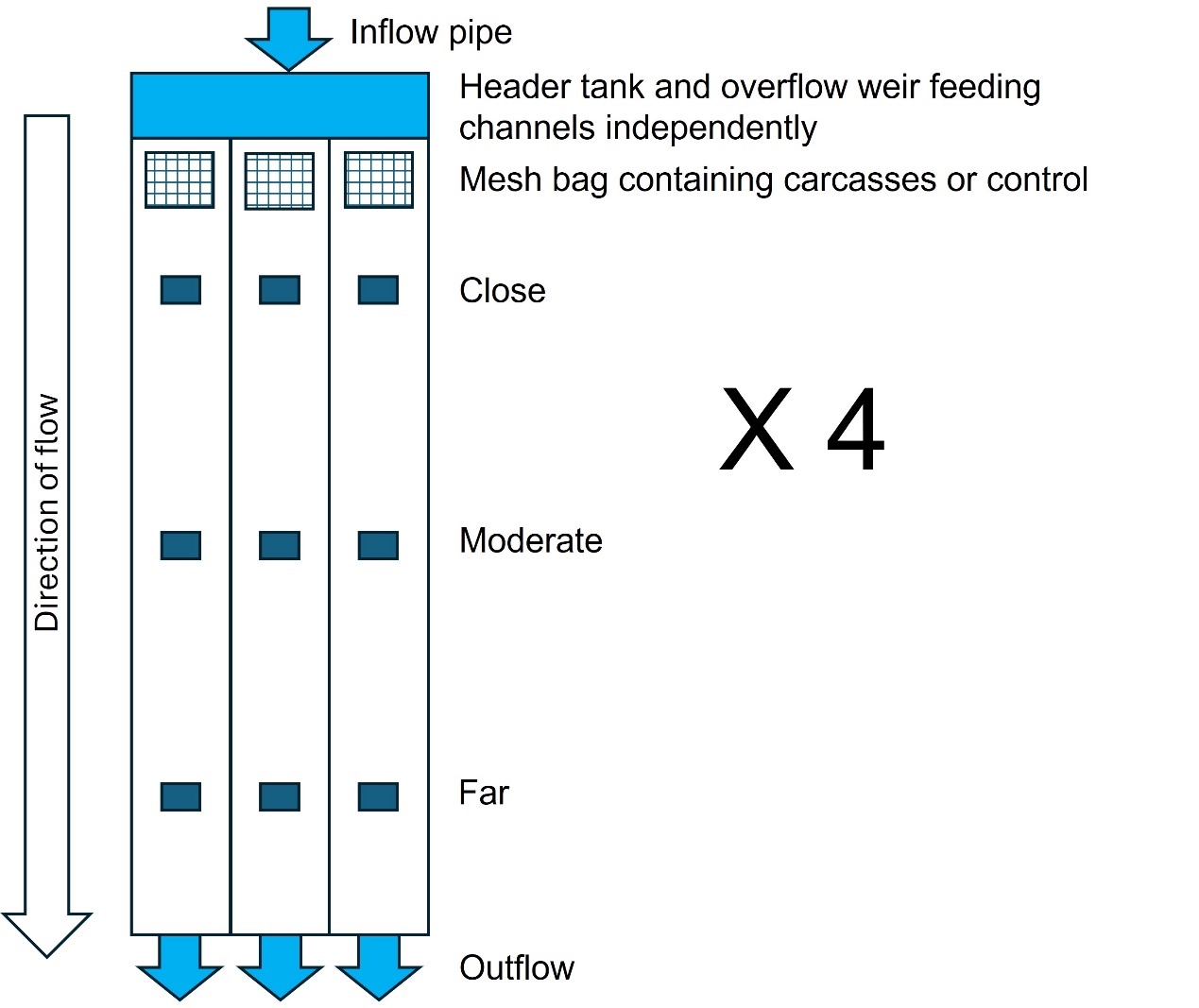


Figure S1 Schematic diagram of the experimental channels showing one block of three channels. Three random surber samples were collected from each on each occasion, either close (1 m), moderate (5 m) or far (10 m) from the mesh bag containing either Control (0 kg m^–2^), Low (0.05 kg m^–2^) or High (0.15 kg m^–2^) loading of carcasses. Four blocks were used, comprising 12 channels in total.

**Table S1** Macroinvertebrate taxa collected in the channel experiment. Specimens were identified to the lowest practical taxonomic level (usually species).

| Family | Species | Functional Feeding group |
| --- | --- | --- |
| Aphelocheiridae | *Aphelocheirus aestivalis* | piercer |
| Planorbidae | *Ancylus fluviatilis* | scraper |
| Athericidae | *Atherix ibis* | piercer |
| Asellidae | *Asellus aquaticus* | shredder |
| Planorbidae | *Anisus vortex* | scraper |
| Brachycentridae | *Brachycentrus fluvialis* | filter feeder |
| Baetidae | *Baetis scambus*/*fuscatus* Lv. | deposit feeder |
| Bithyniidae | *Bithynia tentaculata* | filter feeder |
| Chironomidae | — | deposit feeder |
| Calopterygidae | *Calopteryx splendens* | predator |
| Dytiscidae | — | predator |
| Ephemeridae | *Ephemera danica* | deposit feeder |
| Erpobdellidae | Erpobdella sp | predator |
| Glossiphoniidae | *Glossiphonia complanata* | piercer |
| Gammaridae | *Gammarus pulex* | shredder |
| Hydropsychoidea | *Hydropsyche pellucidula* | filter feeder |
| Hydropsychoidea | *Hydropsyche siltalai* | filter feeder |
| Lymnaeidae | *Lymnaea stagnalis* | scraper |
| Lumbricidae | — | absorber |
| Elmidae | *Limnius volckmari* | shredder |
| Oligochaeta | — | absorber |
| Piscicolidae | *Piscicola geometra* | piercer |
| Polycentropodidae | *Polycentropus Kingi* | predator |
| Limnephilidae | *Potamophylax latipennis* | shredder |
| Lymnaeidae | *Radix balthica* | scraper |
| Rhyacophilidae | *Rhyacophila dorsalis* | predator |
| Ephemerellidae | *Serratella ignita* | deposit feeder |
| Sialidae | *Sialis lutaria* | predator |
| Sericostomatidae | *Sericostoma personatum* | shredder |
| Sphaeriidae | Sphaerium sp | filter feeder |
| Tabanidae |  | shredder |
| Neritidae | *Theodoxus fluviatilis* | scraper |
| Tipulidae | Tipula sp | deposit feeder |

**Table S2** Results of repeated measures general linear mixed model for the impacts of carcasses addition on density and total mass of different functional group and dominant species (n > 200). *** *p* ≤ 0.001, ** *p* ≤ 0.01, * *p* ≤ 0.05 and † *p* ≤ 0.1. TRT= Treatment, DIS= Distance and OCA= Occasion.

| Variable | Functional group/Species | Block | TRT | DIS | TRT*DIS | OCA | TRT*OCA | DIS*OCA | TRT*DIS*OCA |
| --- | --- | --- | --- | --- | --- | --- | --- | --- | --- |
|  |  |  |  |  |  |  |  |  |  |
| lg(Density+1) | Absorber | 1.39 | 0.56 | 0.41 | 0.93 | 3.97** | 1.08 | 0.68 | 0.8 |
|  | Collector-gather | 0.89 | 1.43 | 0.26 | 1.86 | 6.37*** | 0.89 | 2.06 | 0.99 |
|  | Deposit feeder | 6.02*** | 0.01 | 0.80 | 0.32 | 3.94** | 0.13 | 0.57 | 0.35 |
|  | Filter feeder | 8.05*** | 0.80 | 1.70 | 0.09 | 1.12 | 0.14 | 0.51 | 0.34 |
|  | Piercer | 1.81 | 0.46 | 0.44 | 0.36 | 5.54*** | 0.34 | 1.09 | 1.12 |
|  | Predator | 3.66* | 0.75 | 0.43 | 1.06 | 1.45 | 1.24 | 0.32 | 0.95 |
|  | Scraper | 0.49 | 0.78 | 1.58 | 2.05 | 4.16** | 0.59 | 2.71* | 1.24 |
|  | Shredder | 8.36*** | 0.06 | 1.81 | 1.06 | 0.74 | 0.29 | 0.04 | 0.27 |
|  |  |  |  |  |  |  |  |  |  |
| ln(Total mass+1) | Absorber | 0.06 | 1.43 | 0.79 | 0.58 | 1.16 | 0.79 | 0.79 | 0.72 |
|  | Deposit feeder | 2.92* | 0.98 | 0.65 | 1.12 | 1.12 | 0.52 | 0.76 | 0.48 |
|  | Filter feeder | 5.16** | 0.16 | 2.01 | 0.06 | 1.07 | 0.23 | 0.31 | 0.47 |
|  | Piercer | 0.38 | 1.41 | 0.05 | 0.89 | 0.87 | 1.22 | 1.78 | 1.45 |
|  | Predator | 0.54 | 1.00 | 1.35 | 1.16 | 1.66 | 0.43 | 1.24 | 0.63 |
|  | Scraper | 0.79 | 0.58 | 0.35 | 0.51 | 11.88*** | 0.14 | 0.81 | 0.77 |
|  | Shredder | 8.01*** | 0.17 | 4.69** | 2.14 | 2.14 | 0.23 | 0.20 | 0.59 |
|  |  |  |  |  |  |  |  |  |  |
| lg(Density+1) | *Gammarus pulex* | 39.35*** | 0.58 | 4.08† | 1.95 | 1.54 | 0.59 | 0.73 | 0.88 |
|  | Chironomidae | 9.23*** | 1.04 | 1.11 | 0.71 | 37.34*** | 0.56 | 1.05 | 0.72 |
|  | *Ephemera danica* | 8.9*** | 0.14 | 6.28* | 1.52 | 12.67*** | 0.87 | 0.63 | 1.4 |
|  | *Hydropsyche pellucidula* | 17.02*** | 0.1 | 2.88 | 0.55 | 3.92* | 0.56 | 1.25 | 1.18 |
|  | *Asellus aquaticus* | 24.35*** | 0.49 | 6.05* | 3.60* | 7.04*** | 1.35 | 1.22 | 2.17* |
|  |  |  |  |  |  |  |  |  |  |
| ln(Total mass+1) | *Gammarus pulex* | 29.02*** | 1.69 | 3.83† | 1.91 | 1.65 | 0.54 | 0.98 | 0.89 |
|  | Chironomidae | 7.91*** | 1.65 | 3.66† | 0.76 | 2.37 | 0.98 | 0.88 | 0.97 |
|  | *Ephemera danica* | 3.48* | 0.12 | 16.33*** | 1.22 | 17.08*** | 0.72 | 1.48 | 1.92 |
|  | *Hydropsyche pellucidula* | 7.42*** | 0.54 | 2.39 | 0.35 | 2.22 | 0.48 | 0.42 | 0.93 |
|  | *Asellus aquaticus* | 17.14*** | 0.18 | 4.23† | 4.49* | 8.07*** | 1.13 | 0.95 | 2.21* |

**Table S3** Results of the repeated measures general linear mixed model for the impact of block, distance, occasion and treatment, and their interactions on individual mass of macroinvertebrates and the five most abundant species. *** *p* ≤ 0.001, ** *p* ≤ 0.01, * *p* ≤ 0.05 and † *p* ≤ 0.1. TRT= Treatment, DIS= Distance and OCA= Occasion.

| Factors | All macroinvertebrates | *Gammarus pulex* | Chironomidae | *Ephemera danica* | *Hydropsyche pellucidula* | *Asellus aquaticus* |
| --- | --- | --- | --- | --- | --- | --- |
| Block | 1.32 | 177.65*** | 4.38* | 1.66 | 2.73† | 3.18† |
| Species | 47.8*** | / | / | / | / | / |
| OCA | 27.54*** | 54.78*** | 126.58*** | 7.34*** | 1.87 | 2.26 |
| OCA*Species | 21.34*** | / | / | / | / | / |
| TRT | 4.57* | 34.71*** | 3.31 | 4.88* | 2.167 | 0.87 |
| TRT*Species | 2.93*** | / | / | / | / | / |
| OCA*TRT | 8.46*** | 21.36*** | 4.03** | 1.02 | 1.37 | 0.28 |
| OCA*TRT*Species | 10.39*** |  |  |  |  |  |
| DIS | 4.33* | 7.39** | 6.49** | 0.24 | 0 | 0.52 |
| DIS*Species | 1.55** | / | / | / | / | / |
| OCA * DIS | 9.00*** | 13.71*** | 2.37 | 2.12 | 1.71 | 0.52 |
| OCA * DIS *Species | 7.49*** | / | / | / | / | / |
| TRT* DIS | 2.60† | 20.01*** | 2.00 | 1.22 | 1.19 | 0.38 |
| TRT * DIS *Species | 6.61*** |  |  |  |  |  |
| OCA * TRT * DIS | 8.07*** | 15.55*** | 3.03** | 1.72 | 1.16 | 0.89 |
| OCA * TRT * DIS *Species | 5.33*** | / | / | / | / | / |
| Replication | 0.77 | 5.41*** | 0.71 | 2.18*** | 0.98 | 0.92 |

**Table S4** Result of partial redundancy analysis of the impact of pink salmon carcass addition and sampling occasion on the community structure of macroinvertebrates.

| Source | df | Total SS | F- | *p* |
| --- | --- | --- | --- | --- |
| Block | 3 | 0.162 | —— | —— |
| Treatment | 2 | 0.021 | 0.970 | 0.499 |
| Occasion | 3 | 0.398 | 12.369 | 0.001 |
| Treatment*Occasion | 6 | 0.058 | 0.893 | 0.786 |
| Residual | 36 | 0.360 | —— | —— |

**Table S5** Results of repeated-measures general linear mixed models assessing the impacts of pink salmon carcass addition (Treatment) on the stable isotope ratios of carbon (δ^13^C) and nitrogen (δ^15^N) of all macroinvertebrates collectively, macroinvertebrate functional groups (absorber, deposit feeder, filter feeder, piercer, predator, scraper and shredder) and primary producer (*Fontinalis antipyretica* and periphyton). Block, treatment, sampling occasion (Occasion) and distance from the carcasses (Distance) were included in the models as fixed effects and taxon, which was nested at the lowest level, as a random effect. F values are presented with significant results indicated as *** *p* ≤ 0.001, ** *p* ≤ 0.01, * *p* ≤ 0.05 and † *p* ≤ 0.1. Missing values are due to unbalanced sample sizes. TRT= Treatment, DIS= Distance and OCA= Occasion

|  | Isotope | Block | TRT | DIS | TRT*DIS | OCA | TRT*OCA | DIS*OCA | TRT*DIS*OCA | Species(TRT*DIS*OCA) |
| --- | --- | --- | --- | --- | --- | --- | --- | --- | --- | --- |
| **Macroinvertebrates** |  |  |  |  |  |  |  |  |  |  |
| All macroinvertebrates | δ^13^C | 9.31*** | 0.48 | 17.53*** | 2.06† | 62.35*** | 4.58*** | 3.90*** | 3.28*** | 7.78*** |
|  | δ^15^N | 3.32* | 13.07*** | 3.52* | 2.58* | 269.85*** | 2.96** | 1.08 | 3.43*** | 6.54*** |
| Absorbers | δ^13^C | 5.59† | 1.00 | 0.13 | 0.15 | 1.84 | 2.28 | 1.81 | / | 0.10 |
|  | δ^15^N | 5.98† | 0.73 | 3.39 | 1.59 | 0.22 | 1.34 | 4.47 | / | 5.4† |
| Deposit feeders | δ^13^C | 7.39*** | 1.78 | 6.11* | 0.73 | 22.76*** | 1.00 | 1.27 | 1.07 | 2.40*** |
|  | δ^15^N | 0.50 | 16.81*** | 10.57*** | 3.39** | 81.57*** | 21.85*** | 7.15*** | 15.44*** | 30.34*** |
| Filter feeders | δ^13^C | 1.93 | 9.91*** | 11.56*** | 1.47 | 39.05*** | 0.77 | 0.56 | 5.48*** | 12.21*** |
|  | δ^15^N | 1.24 | 1.26 | 1.32 | 1.83 | 41.91*** | 1.8 | 1.26 | 2.05 | 4.88*** |
| Piercers | δ^13^C | 1.08 | 1.40 | 6.49* | 0.80 | 2.35 | 0.46 | 1.45 | 3.98* | 5.95*** |
|  | δ^15^N | 2.01 | 2.99 | 1.58 | 0.67 | 15.19*** | 0.78 | 2.77 | 1.70 | 1.58 |
| Predators | δ^13^C | 3.13 | 5.90† | 1.20 | 2.07 | 16.46*** | 1.62 | 1.87 | 0.49 | 2.46* |
|  | δ^15^N | 0.16 | 0.38 | 0.24 | 0.15 | 10.07*** | 0.85 | 0.79 | 2.87 | 2.26 |
| Scraper | δ^13^C | 6.25** | 2.21 | 5.04* | 0.83 | 3.68* | 1.68 | 3.04† | 0.71 | 3.17*** |
|  | δ^15^N | 2.55 | 5.08* | 0.22 | 0.32 | 71.07*** | 2.92† | 1.43 | 1.69 | 1.64† |
| Shredder | δ^13^C | 3.74† | 0.50 | 11.00*** | 1.35 | 40.32*** | 1.74 | 5.33*** | 1.67 | 6.26*** |
|  | log(δ^15^N) | 0.36 | 1.58 | 2.26 | 2.12† | 74.12*** | 0.78 | 2.13† | 1.80† | 1.94*** |
| **Primary producer** |  |  |  |  |  |  |  |  |  |  |
| *Fontinalis antipyretica* | δ^13^C | 6.10** | 3.12† | 0.10 | 0.13 | 0.37 | 3.03* | 0.07 | 0.11 | / |
|  | log(δ^15^N) | 10.94*** | 4.72* | 0.84 | 0.10 | 4.43* | 1.71 | 0.17 | 0.28 | / |
| Periphyton | δ^13^C | 57.90*** | 7.98** | 0.05 | 0.16 | 1.39 | 4.25** | 0.15 | 0.17 | / |
|  | log(δ^15^N) | 5.08** | 0.41 | 1.50 | 0.89 | 19.73*** | 0.51 | 0.73 | 0.62 | / |

**Table S6** General linear model results for the impacts of pink salmon carcass addition (Treatment) on the stable isotope values of carbon (δ^13^C) and nitrogen (δ^15^N) of the most abundant five species. Block, sampling occasion (Occasion) and distance to the carcasses (Distance) were included in the models. Significant effects in bold. *** *p* ≤ 0.001, ** *p* ≤ 0.01 and * *p* ≤ 0.05. Missing values are due to unbalanced sample sizes. TRT= Treatment, DIS= Distance and OCA= Occasion

| Species | Isotope | Block | TRT | DIS | TRT*DIS | OCA | TRT*OCA | DIS*OCA | TRT*DIS*OCA |
| --- | --- | --- | --- | --- | --- | --- | --- | --- | --- |
| *Gammarus pulex* | δ^13^C | **4.17*** | 1.61 | 1.23 | 1.02 | **39.25***** | 1.02 | 1.22 | 0.55 |
|  | δ^15^N | **5.31**** | 1.03 | 0.53 | 0.14 | **113.81***** | 0.83 | 0.29 | 1.26 |
|  |  |  |  |  |  |  |  |  |  |
| Chironomidae | δ^13^C | 2.22 | 2.66 | 0.48 | 1.81 | 1.13 | 0.88 | 1.37 | / |
|  | δ^15^N | 1.77 | 0.19 | 0.95 | 3.04 | 0.43 | 4.65 | 4.01 | / |
|  |  |  |  |  |  |  |  |  |  |
| *Ephemera Danica* | δ^13^C | **7.70 ***** | 0.62 | 3.29 | 1.64 | **12.11***** | 1.09 | 1.37 | 0.69 |
|  | δ^15^N | 1.22 | 2.22 | 1.72 | 0.96 | **24.28***** | 1.12 | 0.96 | 1.08 |
|  |  |  |  |  |  |  |  |  |  |
| *Asellus aquaticus* | δ^13^C | **6.95***** | 0.55 | **11.96***** | **5.86***** | 0.55 | 2.10 | 0.73 | 1.38 |
|  | δ^15^N | 2.09 | 0.30 | **4.60*** | 0.58 | 0.28 | 1.28 | 1.13 | 1.69 |
|  |  |  |  |  |  |  |  |  |  |
| *Hydropsyche pellucidula* | δ^13^C | 1.25 | 1.18 | **6.41**** | 0.76 | **10.65***** | 1.76 | 0.57 | 1.22 |
|  | δ^15^N | 0.90 | 1.51 | 0.92 | 2.08 | **17.76***** | 1.23 | 0.17 | 1.89 |

**Table S7** Repeated-measures general linear mixed model results for assessing the impacts of pink salmon carcass addition (Treatment) on elemental content (%C and %N) and ratio (C:N) of macroinvertebrates, functional groups and basal resources in the experimental channels. Block, treatment, and distance from the carcasses (Distance) were included in the models as fixed effect and taxon, which was nested at the lowest level, as a random effect. F values are presented with significant results indicated as *** *p* ≤ 0.001, ** *p* ≤ 0.01, * *p* ≤ 0.05 and † *p* ≤ 0.1. Missing values for the interaction term were indicated the values were not calculated due to unbalanced sample sizes. The abbreviations are: TRT= Treatment, DIS= Distance and OCA= Occasion

|  | Nutrient content | Block | TRT | DIS | TRT*DIS | OCA | TRT*OCA | DIS*OCA | TRT*DIS*OCA | Species(TRT*DIS*OCA) |
| --- | --- | --- | --- | --- | --- | --- | --- | --- | --- | --- |
| **Macroinvertebrates** |  |  |  |  |  |  |  |  |  |  |
| All macroinvertebrates | C | 0.47 | 3.36* | 0.82 | 3.20* | 4.35** | 0.61 | 1.15 | 1.37 | 3.12*** |
|  | N | 0.16 | 2.97† | 1.70 | 3.31* | 1.96 | 1.02 | 0.59 | 1.05 | 3.21*** |
|  | Ln(C:N) | 0.78 | 0.08 | 3.21* | 1.51 | 3.19* | 1.37 | 1.07 | 0.89 | 2.35*** |
| Absorber | C | 1.28 | 0.29 | 0.05 | 0.37 | 1.66 | 0.39 | 0.21 | / | 1.75 |
|  | N | 0.81 | 0.67 | 0.05 | 0.52 | 2.29 | 0.46 | 0.55 | / | 0.77 |
|  | Ln(C:N) | 0.58 | 0.88 | 0.19 | 0.45 | 1.04 | 0.33 | 1.07 | / | 4.50 |
| Deposit feeder | C | 6.80*** | 0.20 | 1.25 | 0.55 | 14.88*** | 0.81 | 0.79 | 0.41 | 1.25 |
|  | N | 6.49** | 0.82 | 1.64 | 0.66 | 12.04*** | 0.51 | 0.79 | 0.54 | 1.42 |
|  | Ln(C:N) | 0.11 | 4.40† | 0.71 | 4.05* | 2.81 | 2.93† | 0.94 | 0.59 | 2.28 |
| Filter feeder | C | 0.49 | 3.34* | 2.77 | 9.37*** | 8.23*** | 1.00 | 1.74 | 2.51† | 7.24*** |
|  | N | 1.19 | 2.68 | 3.57 | 8.21*** | 3.21 | 1.08 | 1.06 | 2.84* | 9.28*** |
|  | Ln(C:N) | 2.01 | 0.79 | 0.72 | 0.89 | 3.20 | 0.46 | 0.54 | 1.89 | 0.77 |
| Piercer | C | 1.88 | 0.02 | 0.93 | 0.28 | 0.24 | 1.30 | 0.62 | 0.37 | 0.70 |
|  | N | 0.78 | 0.08 | 0.16 | 0.33 | 0.51 | 1.37 | 0.72 | 0.26 | 0.52 |
|  | Ln(C:N) | 0.75 | 0.10 | 1.24 | 0.93 | 1.04 | 0.14 | 0.67 | 0.51 | 1.11 |
| Predator | C | 1.03 | 0.39 | 0.24 | 0.65 | 0.50 | 0.60 | 0.29 | 0.41 | 0.96 |
|  | N | 0.80 | 0.34 | 1.26 | 0.45 | 1.28 | 0.48 | 0.44 | 0.30 | 1.65 |
|  | Ln(C:N) | 1.23 | 0.40 | 0.76 | 0.46 | 0.94 | 0.55 | 1.45 | 0.47 | 0.98 |
| Scraper | C | 4.21* | 0.73 | 0.59 | 0.73 | 1.10 | 0.39 | 0.44 | 1.99 | 0.72 |
|  | N | 3.69† | 0.65 | 0.86 | 0.47 | 1.10 | 0.51 | 0.37 | 2.21† | 0.64 |
|  | Ln(C:N) | 0.14 | 0.01 | 0.86 | 0.22 | 1.07 | 0.82 | 0.16 | 0.98 | 0.95 |
| Shredder | C | 1.27 | 2.26 | 0.91 | 2.41 | 29.89*** | 3.37* | 4.47** | 6.09*** | 11.15*** |
|  | N | 1.60 | 0.91 | 0.48 | 2.46 | 14.95*** | 1.85 | 1.46 | 2.35* | 5.66*** |
|  | Ln(C:N) | 1.42 | 2.16 | 1.97 | 2.28 | 2.32 | 0.40 | 3.03† | 1.67 | 6.01*** |
| **Primary producer** |  |  |  |  |  |  |  |  |  |  |
| Periphyton | C | 11.20*** | 5.13* | 0.25 | 0.06 | 29.11*** | 1.74 | 0.16 | 0.35 | / |
|  | N | 13.33*** | 1.45 | 0.17 | 0.07 | 58.10*** | 1.17 | 0.13 | 0.22 | / |
|  | Ln(C:N) | 18.17*** | 1.51 | 0.26 | 0.19 | 36.08*** | 1.03 | 0.09 | 0.19 | / |
| *Fontinalis antipyretica* | C | 5.07** | 0.02 | 0.05 | 0.05 | 8.32*** | 0.11 | 0.06 | 0.09 | / |
|  | N | 5.74** | 0.33 | 0.16 | 0.16 | 8.24*** | 0.34 | 0.04 | 0.30 | / |
|  | Ln(C:N) | 3.58* | 2.91 | 1.55 | 0.66 | 9.78*** | 1.08 | 0.15 | 0.57 | / |
